# PD-L1–stratified health-related quality of life in solid tumors: pembrolizumab versus chemotherapy—a narrative review

**DOI:** 10.3389/fmed.2026.1890061

**Published:** 2026-07-24

**Authors:** Eman Shorog, Lavanya Selvaraj, Geetha Kandasamy, Tariq Eidhah Alzahrani, Alhanouf F. Almousa, Maram A. Alenazi, Hanan Alshareef, Shanthanalakshmi Siva Basavan

**Affiliations:** 1 Department of Clinical Pharmacy, College of Pharmacy, King Khalid University, Abha, Saudi Arabia; 2 Department of Pharmacy Practice, PSG College of Pharmacy, Coimbatore, Tamil Nadu, India; 3 Saudi Society of Clinical Pharmacy, Riyadh, Saudi Arabia; 4 King Faisal Specialist Hospital and Research Centre, Riyadh, Saudi Arabia; 5 Pharmaceutical Care Services, King Salman Specialist Hospital, Hail Health Cluster, Ministry of Health, Hail, Saudi Arabia; 6 Department of Pharmacy Practice, Faculty of Pharmacy, University of Tabuk, Tabuk, Saudi Arabia

**Keywords:** chemotherapy, health-related quality of life, immunotherapy, PD-L1, pembrolizumab, solid tumors

## Abstract

The introduction of immune checkpoint inhibitors has transformed therapeutic strategies for multiple solid malignancies, with pembrolizumab emerging as a key treatment option. It is used as monotherapy in patients with high programmed death-ligand 1 (PD-L1) expression and in combination with chemotherapy in broader clinical settings. Although survival benefits are well established, understanding the impact of these treatment approaches on health-related quality of life (HRQoL) is essential for long-term cancer care. This review aims to summarize and compare HRQoL outcomes in patients with solid tumors receiving pembrolizumab monotherapy versus combination chemotherapy, while considering disease-specific PD-L1 scoring systems (tumor proportion score [TPS] and combined positive score [CPS]) and expression thresholds to support personalized treatment strategies. This narrative review synthesized evidence from randomized controlled trials, observational studies, meta-analyses, and real-world analyses identified through literature searches of major databases, including PubMed, Scopus, and Google Scholar, focusing on studies reporting HRQoL outcomes in patients receiving pembrolizumab-based therapies. A total of 64 studies were included in this narrative review. Across the included studies, HRQoL findings were heterogeneous because of differences in assessment instruments, follow-up time points, and endpoint definitions. Pembrolizumab monotherapy was commonly associated with HRQoL maintenance, delayed deterioration, or improvement in selected HRQoL domains, particularly in disease settings where pembrolizumab monotherapy is indicated for patients with higher PD-L1 expression. In contrast, combination chemotherapy was frequently associated with an early decline in HRQoL attributable to treatment-related toxicity, followed by stabilization or partial recovery, reflecting long-term functional preservation in patients achieving disease control. Overall, HRQoL outcomes varied according to tumor type, PD-L1 scoring framework, baseline symptom burden, assessment timing, and the specific HRQoL endpoint evaluated. Pembrolizumab-based therapy plays a pivotal role in modern oncology by improving survival while maintaining favorable HRQoL outcomes in appropriately selected patients. However, direct comparisons between pembrolizumab monotherapy and combination chemotherapy should be interpreted cautiously because differences in HRQoL instruments, assessment timing, endpoint definitions, and disease-specific PD-L1 assessment methodologies may contribute to the observed findings. Disease-specific PD-L1-guided treatment selection may help balance therapeutic efficacy with patient well-being; however, HRQoL findings should be interpreted within tumor-specific PD-L1 frameworks rather than as evidence of a uniform PD-L1-driven effect across all solid tumors. Future research should prioritize prospective head-to-head HRQoL comparisons using standardized HRQoL instruments, harmonized endpoint definitions, consistent assessment schedules, and disease-specific PD-L1-stratified analyses across tumor types to optimize patient-centered treatment strategies.

## 1 Introduction

Immunotherapy has transformed oncology by harnessing the immune system to target and eliminate cancer cells. Among immune checkpoint inhibitors, pembrolizumab (anti-PD-1 monoclonal antibody) has become a cornerstone therapy for several solid tumors, including non-small cell lung cancer (NSCLC), gastric cancer, and head and neck squamous cell carcinoma (HNSCC) (1–4). By blocking the interaction between PD-1 receptors and programmed death-ligand 1 (PD-L1), pembrolizumab restores T-cell activation and enhances antitumor immune responses (5–7), and has been associated with durable clinical responses and improved long-term survival in multiple clinical studies (1–4). While MSI-H/dMMR represents a highly specific predictor of pembrolizumab benefit driven by defective mismatch repair and high neoantigen burden its clinical utility is limited by low prevalence across solid tumors (8). PD-L1 expression, assessed by immunohistochemistry, has received FDA approval as a companion diagnostic for pembrolizumab across multiple tumor types including NSCLC, gastric, cervical, and head and neck cancers, making it the most broadly implemented biomarker in routine oncology practice (1, 9). Given its central role in stratifying patients for pembrolizumab monotherapy versus combination chemotherapy, this narrative review focuses on PD-L1 expression as the primary biomarker to examine the differential impact on health-related quality of life across solid tumors.

In clinical practice, treatment selection is often guided by PD-L1 expression. Pembrolizumab monotherapy is preferred for patients with high PD-L1 levels (e.g., ≥50% tumor proportion score [TPS] in NSCLC), offering effective disease control with relatively low toxicity (1, 10). In contrast, combination therapy with chemotherapy is frequently employed in patients with lower PD-L1 expression or additional clinical considerations, providing synergistic benefits but potentially increasing treatment-related adverse events (11–13).

While clinical trials have demonstrated survival benefits, the impact on health-related quality of life (HRQoL) including physical, psychological, and social functioning has received growing attention. Patient-reported outcome measures (PROs), such as the European Organisation for Research and Treatment of Cancer Quality of Life Questionnaire-Core 30 (EORTC QLQ-C30) and the Functional Assessment of Cancer Therapy (FACT) scales, are validated instruments commonly used to assess symptom burden, functional ability, and overall well-being in oncology populations (14, 15). According to the U.S. Food and Drug Administration (FDA) Guidance for Industry (2018), clinical benefit in oncology trials includes symptomatic improvement, which may be evaluated using patient-reported outcomes (PROs) as important efficacy endpoints (16). Evidence suggests that treatment-related toxicities (e.g., fatigue, nausea, and immune-mediated adverse events) may temporarily reduce HRQoL in combination regimens, whereas monotherapy often preserves or improves quality of life in selected patients (17–19). This narrative review synthesizes evidence from randomized trials, observational studies, and real-world data to compare HRQoL outcomes in patients receiving pembrolizumab monotherapy versus combination chemotherapy, stratified by PD-L1 expression, with the aim of informing personalized treatment strategies and guiding therapy selection to optimize both survival and patient-centered outcomes. By focusing on pivotal trials including KEYNOTE-024, KEYNOTE-042, KEYNOTE-189, KEYNOTE-062, and KEYNOTE-048, the findings are intended to support treatment decisions that balance clinical benefits with patient quality of life (1–4, 10).

## 2 Methods

### 2.1 Study design

This narrative review was conducted to synthesize evidence on PD-L1–stratified health-related quality of life (HRQoL) outcomes in patients with solid tumors receiving pembrolizumab monotherapy versus pembrolizumab combined with chemotherapy. A comprehensive literature search was performed using PubMed, Scopus, and Google Scholar for articles published between January 2015 and May 2026 to capture relevant studies following the clinical introduction of pembrolizumab.

Search terms included combinations of “pembrolizumab,” “PD-L1,” “health-related quality of life,” “HRQoL,” “solid tumors,” “chemotherapy,” “immunotherapy,” and specific trial names (e.g., KEYNOTE-024 and KEYNOTE-048). A representative PubMed search strategy included the following search syntax: (“Pembrolizumab” AND “PD-L1”) AND (“Health-Related Quality of Life” OR HRQoL OR “Patient-Reported Outcomes”) AND (“Solid Tumors” OR cancer). Equivalent search strategies were adapted for Scopus and Google Scholar according to the indexing requirements of each database. Database-specific search strategies were adapted according to the indexing requirements of each database using Boolean operators (AND/OR). For PubMed, combinations of Medical Subject Headings (MeSH), where applicable, and free-text terms were used, while equivalent keyword-based search strategies were applied in Scopus and Google Scholar. Reference lists of all included studies and relevant review articles were manually screened to identify additional eligible publications.

### 2.2 Inclusion and exclusion criteria

Included studies were peer-reviewed randomized controlled trials, observational studies, meta-analyses, cross-sectional studies, and real-world analyses reporting PROs or HRQoL outcomes in patients receiving pembrolizumab monotherapy or pembrolizumab combined with chemotherapy, with reported PD-L1 expression levels (e.g., ≥1%, ≥20%, or ≥50% using tumor proportion score [TPS] or combined positive score [CPS]). Studies were excluded if they were non-English publications, editorials, opinion pieces without original data, conference abstracts, non-peer-reviewed articles, grey literature, single-case reports, preclinical or non-clinical studies, pharmacokinetic/pharmacodynamic-only studies, or articles lacking HRQoL outcomes, PD-L1 stratification, or relevant treatment comparisons.

### 2.3 Study selection and data extraction

A total of 249 records were identified through database searching. After removing duplicate records, two reviewers independently screened the titles and abstracts for relevance to the objectives of this narrative review. Full-text articles were subsequently assessed for eligibility according to the predefined inclusion and exclusion criteria. Any disagreements regarding study selection or data extraction were resolved through discussion until consensus was reached. Studies that did not align with the review objectives, did not report HRQoL outcomes, lacked PD-L1 stratification, or did not involve patients with solid tumors were excluded. Following full-text assessment, 64 studies met the eligibility criteria and were included in the narrative synthesis. Following full-text assessment, 64 studies met the eligibility criteria and were included in the narrative synthesis.

**Figure 1:**
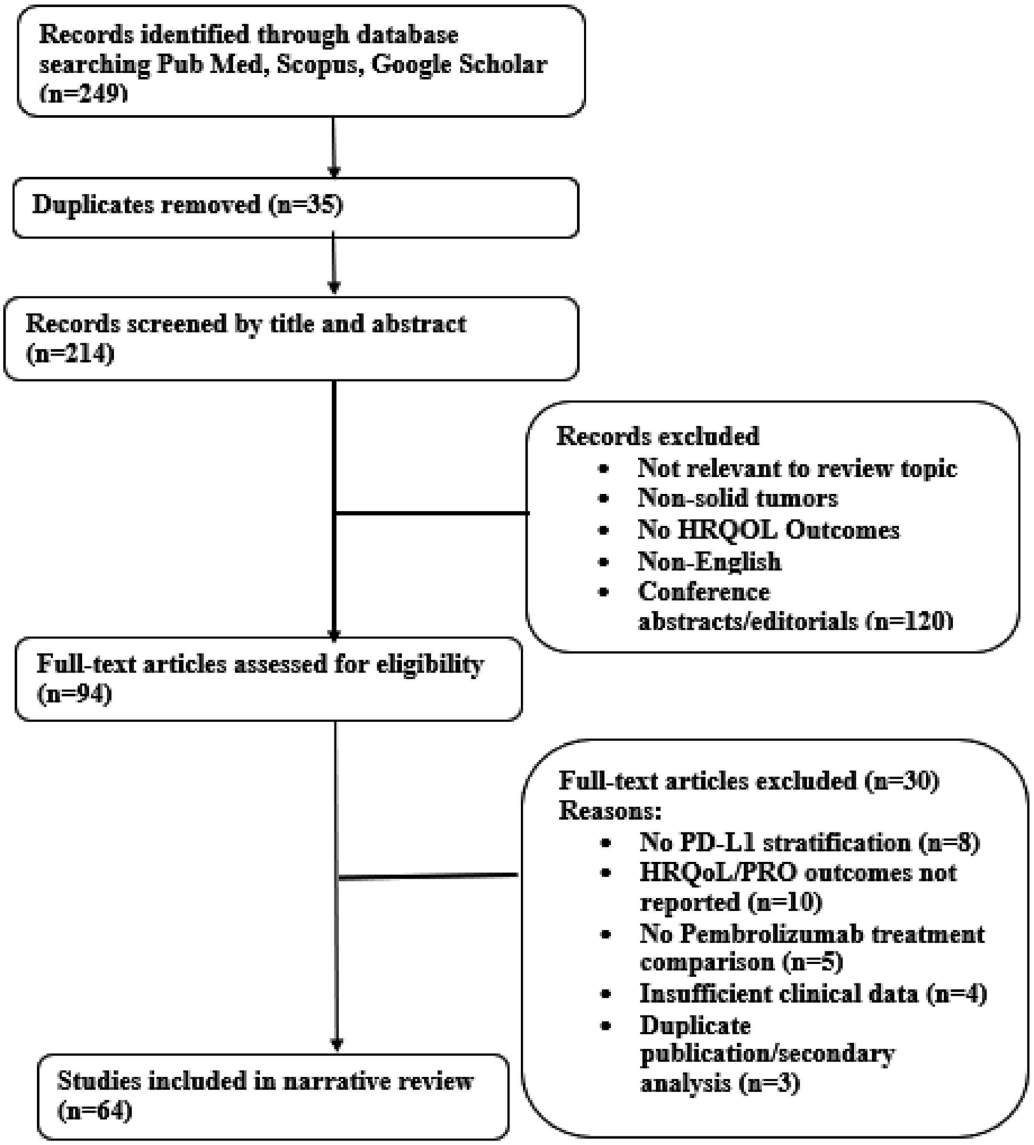
Literature search and study selection process. HRQoL, Health related quality of life; PRO, Patient-reported outcome.

For each included study, the following data were extracted: study design, cancer type, sample size, treatment regimen, PD-L1 expression threshold, HRQoL instrument(s), HRQoL endpoint(s), follow-up duration, and the principal findings relevant to the objectives of this review. As this review was narrative in nature and intended to provide a comprehensive synthesis of the available evidence, a formal methodological quality or risk-of-bias assessment was not performed. To enhance transparency and reproducibility, the literature search and study selection process is summarized in Figure 1 (literature search and study selection process).

### 2.4 Data synthesis

**Figure 2:**
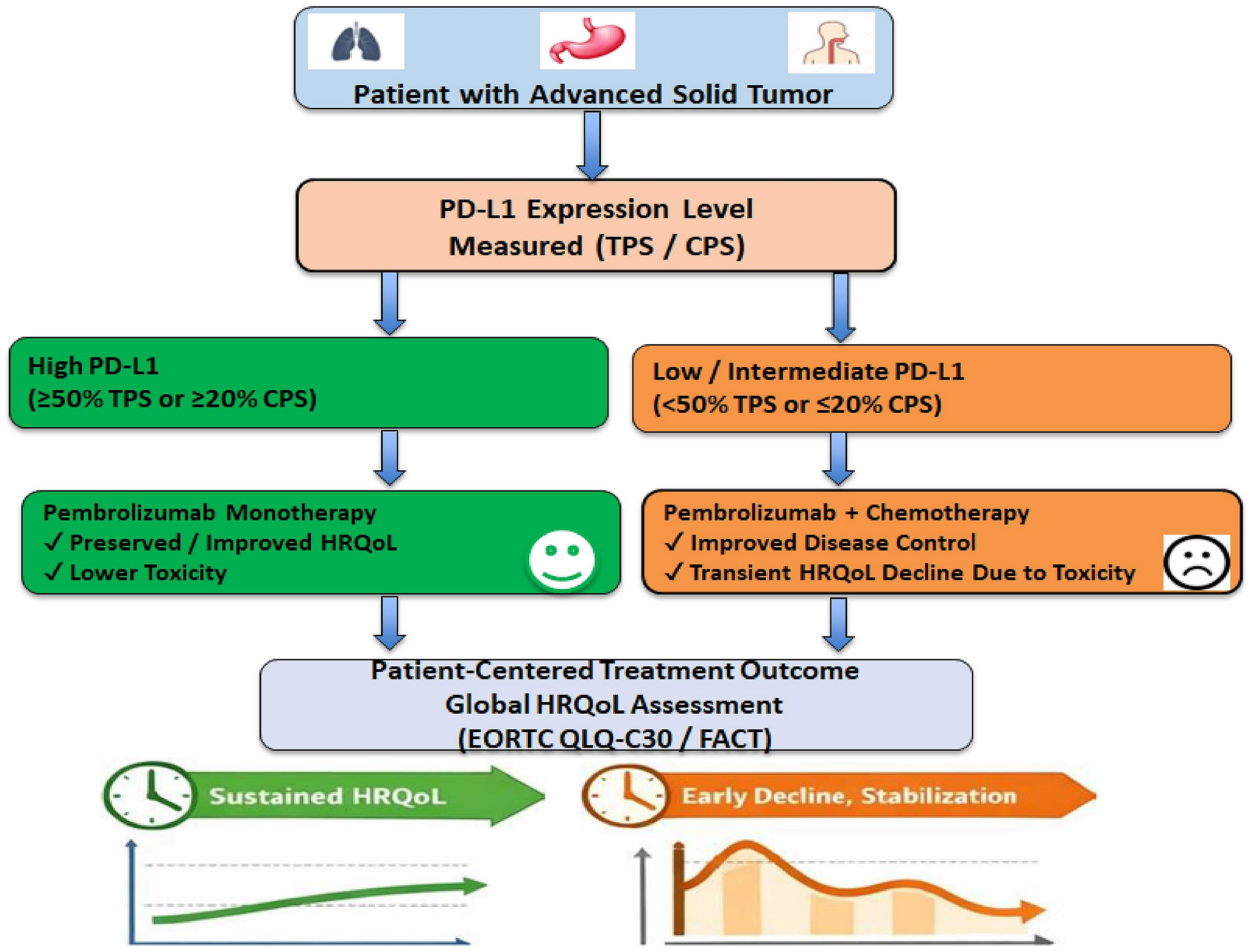
PD-L1–stratified treatment strategy with pembrolizumab and associated HRQoL outcomes. PD-L1, Programmed Death-Ligand 1; TPS, Tumor Proportion Score; CPS, Combined Positive Score; HRQoL, Health-Related Quality of Life; EORTC QLQ-C30, European Organisation for Research and Treatment of Cancer – Quality of Life Questionnaire-Core 30; FACT, Functional Assessment of Cancer Therapy.

Given the heterogeneity of study designs, tumor types, and HRQoL assessment tools, a narrative synthesis approach was employed. Outcomes were analyzed based on PD-L1 expression levels, treatment modality (monotherapy vs. combination), and tumor type to provide insights into personalized treatment strategies (see Figure 2).

## 3 PD-L1–stratified health-related quality of life outcomes

### 3.1 HRQoL according to PD-L1 expression across tumor types

PD-L1 expression, assessed using tumor proportion score (TPS) in lung cancer and CPS in gastrointestinal, cervical, and head and neck cancers, plays a central role in selecting patients for pembrolizumab-based therapy. Because TPS and CPS represent distinct PD-L1 scoring methodologies and are applied using disease-specific positivity thresholds and companion diagnostic assays, direct comparisons of HRQoL outcomes across tumor types should be interpreted cautiously (20). Beyond its established association with survival outcomes, emerging evidence suggests that PD-L1 status may also be associated with differences in HRQoL trajectories during immunotherapy, although the available evidence does not establish a uniform PD-L1-specific effect on HRQoL across all solid tumors, and this aspect has been less systematically evaluated.

Phase III randomized controlled trials evaluating immune checkpoint inhibitors across solid tumors have demonstrated consistent survival benefits, particularly in lung, head and neck, and gastroesophageal malignancies where PD-L1-guided treatment strategies are well established. However, HRQoL outcomes were reported inconsistently across studies, and differences in PD-L1 scoring systems, positivity thresholds, and assessment methodologies further limit direct cross-trial comparisons (20), limiting the ability to perform pooled analyses focused specifically on patient-reported outcomes and highlighting the need for focused evidence synthesis in this area (21).

A systematic review and meta-analysis further demonstrated that PD-1/PD-L1 inhibitor therapy was associated with delayed deterioration in global health status compared with chemotherapy across multiple solid tumors, with mean changes in EORTC QLQ-C30 scores favoring immunotherapy in comparative analyses (22). Similarly, an analysis of 34 randomized trials involving 18,709 patients reported that immunotherapy-based regimens, including monotherapy and combination approaches, were associated with prolonged time to clinically meaningful deterioration in patient-reported outcomes compared with non-immunotherapy controls across several tumor types (23). Although these findings support the overall HRQoL benefit of immune checkpoint inhibitor-based therapy, they should not be interpreted as demonstrating a consistent PD-L1-specific HRQoL effect across different malignancies.

The influence of PD-L1 expression on HRQoL may partly reflect differences in treatment exposure and disease control. Patients with high PD-L1 expression receiving pembrolizumab monotherapy often avoid chemotherapy-related toxicities such as fatigue, nausea, alopecia, and myelosuppression, and this pattern is accompanied by preservation of physical and social functioning. Observational findings suggest that improved tumor control has been reported alongside reduced cancer-related symptom burden and overall well-being (24). However, these associations are also likely influenced by tumor type, treatment strategy, and patient characteristics, rather than PD-L1 expression alone.

In contrast, patients with lower PD-L1 expression frequently receive chemoimmunotherapy combinations, which may be associated with an initial decline in HRQoL during the chemotherapy induction phase because of cumulative treatment-related adverse effects. Nevertheless, HRQoL may subsequently stabilize or improve in the context of disease response and ongoing treatment (25). These differing trajectories provide an important framework for interpreting patient-reported outcomes across pembrolizumab-based treatment strategies stratified by PD-L1 expression, while recognizing that disease-specific PD-L1 scoring frameworks (TPS versus CPS), positivity thresholds, and companion diagnostic assays should be considered when interpreting HRQoL outcomes across different solid tumors (20).

### 3.2 HRQoL with pembrolizumab monotherapy

Across multiple solid tumor indications, pembrolizumab monotherapy has been consistently associated with maintenance or improvement in HRQoL, particularly in PD-L1–selected populations where durable disease control may be achieved with a lower treatment-related symptom burden compared with conventional cytotoxic therapy. These outcomes have been evaluated using global health status measures, functional domain subscales, and time-to-deterioration analyses, most commonly through the EORTC QLQ-C30 instrument used in solid tumor studies (26, 27).

The five-year follow-up analysis from KEYNOTE-024 provides some of the most mature evidence supporting this association. In patients with advanced NSCLC and PD-L1 TPS ≥ 50%, pembrolizumab demonstrated a substantial overall survival advantage over platinum-based chemotherapy (31.9% vs. 16.3%) while showing a stable tolerability profile during prolonged follow-up. Patient-reported outcome analyses showed sustained separation in HRQoL scores between groups over time, with increasing differences during extended follow-up as cumulative toxicity was more frequently reported in the chemotherapy group (28).

Similar findings were observed in gastric and gastroesophageal junction cancers. In KEYNOTE-061, pembrolizumab was associated with maintenance of EORTC QLQ-C30 global health status, physical functioning, and STO22 scores, whereas paclitaxel-treated patients reported worsening fatigue and functional decline. In malignancies associated with substantial baseline symptom burden, preservation of functional status during later-line therapy represents a clinically relevant outcome (29). Comparable HRQoL patterns have also been reported in advanced hepatocellular carcinoma, where pembrolizumab monotherapy was associated with maintenance of global health status compared with best supportive care, while patients receiving placebo showed greater deterioration in quality-of-life measures over time (30).

Additional insights were provided by a model-based meta-analysis using nonlinear mixed-effects modeling of longitudinal EORTC QLQ-C30 global health status data from more than 20 pembrolizumab trials. Although several studies reported limited differences at predefined timepoints, longitudinal modeling demonstrated a consistent pattern in which pembrolizumab-treated patients experienced a mild and transient decline in HRQoL early during therapy, followed by gradual improvement and long-term stabilization. These findings suggest that conventional fixed time-point analyses may underestimate long-term HRQoL patterns associated with immunotherapy (31).

### 3.3 HRQoL with pembrolizumab plus chemotherapy

For patients with lower PD-L1 expression, chemotherapy alone is often insufficient to achieve durable disease control, and pembrolizumab is commonly added as part of first-line treatment. This chemoimmunotherapy approach has become standard across several malignancies, including NSCLC, gastric, esophageal, cervical, head and neck, and triple-negative breast cancers. Unlike pembrolizumab monotherapy, where HRQoL is generally maintained from early treatment onward, patients receiving combination therapy frequently experience an initial decline in HRQoL during chemotherapy induction, characterized by fatigue, nausea, appetite loss, and reduced physical functioning. The clinically important question, however, is whether the addition of pembrolizumab is associated with additional HRQoL burden beyond chemotherapy alone. Current evidence suggests that it is not associated with clinically meaningful additional deterioration in HRQoL compared with chemotherapy alone (32–34).

In NSCLC, patient-reported outcome analyses from KEYNOTE-189 and KEYNOTE-407 demonstrated that pembrolizumab plus chemotherapy was associated with maintenance of global health status throughout treatment, without clinically meaningful differences compared with chemotherapy alone. Beyond the induction phase, symptom burden showed trends toward improvement over time, likely reflecting disease control. These studies also demonstrated superior progression-free survival and higher response rates across PD-L1 expression levels, including patients with TPS ≥ 50%, although combination therapy was associated with increased treatment-related adverse events (35).

In the perioperative setting, KEYNOTE-671 demonstrated that the addition of pembrolizumab to neoadjuvant chemotherapy in resectable NSCLC was associated with maintenance of HRQoL during both neoadjuvant and adjuvant phases, supporting tolerability across the treatment continuum (36). Evidence from gynecologic malignancies further supports tolerability of pembrolizumab-based combinations. A review of immune checkpoint inhibitor use in gynecologic cancers reported no major increase in grade 3–4 adverse events compared with chemotherapy alone, while patient-reported outcomes indicated overall preservation of functional status during treatment (37). Similarly, KEYNOTE-826 showed that adding pembrolizumab to platinum-based chemotherapy, with or without bevacizumab, was not associated with clinically meaningful worsening of HRQoL in cervical cancer, including in patients with substantial baseline symptom burden.

Comparable HRQoL patterns were observed in KEYNOTE-355 and KEYNOTE-590. Patients experienced the expected early decline associated with chemotherapy exposure; however, HRQoL subsequently stabilized, and pembrolizumab was not associated with additional reported symptom burden beyond chemotherapy effects during induction therapy. A quality-adjusted survival analysis from KEYNOTE-590 using Q-TWiST methodology demonstrated that pembrolizumab plus chemotherapy provided improved quality-adjusted survival compared with chemotherapy alone in esophageal cancer (38). Likewise, in early-stage triple-negative breast cancer, KEYNOTE-522 showed that neoadjuvant pembrolizumab plus chemotherapy followed by adjuvant pembrolizumab was not associated with adverse HRQoL outcomes compared with chemotherapy alone across both treatment phases (39). Across thoracic, gastrointestinal, gynecologic, and breast cancer settings, available evidence indicates that the addition of pembrolizumab is not associated with substantial additional HRQoL deterioration beyond chemotherapy-related effects. Instead, disease control is commonly reported alongside stabilization or gradual recovery of HRQoL over time. Supporting this, a meta-analysis of immune checkpoint inhibitor-based regimens in gastroesophageal cancers demonstrated maintained or improved HRQoL outcomes with immunotherapy-containing combinations across randomized controlled trials (40).

### 3.4 Comparative outcomes: monotherapy versus combination therapy

When comparing pembrolizumab monotherapy and chemoimmunotherapy from a patient-reported outcomes (PRO) perspective, differences extend beyond antitumor efficacy alone. These treatment strategies follow distinct clinical trajectories with respect to the timing, magnitude, and durability of HRQoL changes. Overall, HRQoL outcomes are influenced by tumor biology, PD-L1 expression status, and the balance between treatment-related toxicity and disease control achieved with each approach. Consequently, HRQoL outcomes should not be interpreted as uniformly superior or inferior across regimens but rather as context-dependent within biomarker-selected populations and treatment intent settings (41). Importantly, comparisons between monotherapy and combination therapy should be interpreted cautiously because these treatment approaches are typically administered to clinically and biologically different patient populations. Patients receiving pembrolizumab monotherapy are generally selected based on high PD-L1 expression and disease-specific treatment indications, whereas chemoimmunotherapy is more commonly used in patients with lower PD-L1 expression, higher disease burden, or more aggressive disease requiring rapid disease control (1–4, 10). Therefore, observed differences in HRQoL may reflect differences in baseline prognosis and patient characteristics in addition to treatment-related effects (41–46).

Direct evidence from biomarker-selected populations demonstrates HRQoL differences between treatment strategies. In KEYNOTE-177, first-line pembrolizumab was associated with maintenance or improvement in HRQoL measures, whereas chemotherapy was associated with deterioration in global health status, functional domains, and symptom scales of the EORTC QLQ-C30. These findings highlight differences in patient-reported outcomes across treatment strategies in MSI-H/dMMR metastatic colorectal cancer (41).

In PD-L1–high NSCLC, long-term follow-up data from KEYNOTE-024 are consistent with durable clinical benefit and favorable tolerability profiles with pembrolizumab monotherapy compared with platinum-based chemotherapy (42). Similarly, KEYNOTE-006 in advanced melanoma demonstrated sustained survival outcomes and continued tolerability of pembrolizumab compared with ipilimumab (43). Although HRQoL was not a primary long-term endpoint, safety and disease control patterns are consistent with sustained functional preservation in responding patients.

Comparative patient-reported outcome evidence suggests that immune checkpoint inhibitor monotherapy is generally associated with maintenance or improvement in HRQoL relative to chemotherapy-based regimens, while combination regimens show intermediate patterns, reflecting partial mitigation of chemotherapy-associated HRQoL decline (44). Real-world evidence in PD-L1–high metastatic lung cancer suggests comparable survival outcomes between pembrolizumab monotherapy and chemoimmunotherapy in selected populations, supporting biomarker-guided treatment selection (45). However, HRQoL data remain limited in these studies. Similarly, in urothelial carcinoma, pembrolizumab monotherapy is associated with improved tolerability compared with cytotoxic chemotherapy, although HRQoL interpretations remain limited due to secondary endpoint status (46). Accordingly, the available evidence should not be interpreted as demonstrating the intrinsic superiority of pembrolizumab monotherapy over combination therapy, as differences in baseline clinical characteristics, disease burden, treatment indication, and prognosis may also contribute to the observed HRQoL outcomes (41–46).

Across treatment strategies, toxicity profiles differ in timing and nature. Pembrolizumab monotherapy is associated with immune-related adverse events such as thyroid dysfunction, dermatitis, and pneumonitis, which are typically delayed in onset and manageable with immunosuppressive strategies. Chemotherapy-related toxicities-including fatigue, nausea, anorexia, and myelosuppression-are typically early-onset and associated with acute functional impairment. In combination regimens, pembrolizumab is not consistently associated with additional HRQoL deterioration beyond chemotherapy-related effects (44). In clinical practice, chemoimmunotherapy is generally preferred for lower PD-L1 expression or aggressive disease biology requiring rapid disease control. In these contexts, HRQoL may initially decline during chemotherapy exposure but may stabilize or improve over time in association with treatment response. In contrast, pembrolizumab monotherapy is associated with more stable long-term HRQoL in PD-L1–high populations, where durable responses may be achieved without chemotherapy exposure (41–43). However, these observations should be interpreted in the context of patient selection, baseline disease characteristics, treatment indication, and prognosis, rather than being attributed solely to treatment effects (41–46). Accordingly, differences in HRQoL observed between treatment strategies should be interpreted within the context of the specific HRQoL endpoint evaluated and the timing of assessment.

## 4 Discussion

### 4.1 Interpretation of findings

The findings from this narrative review highlight the critical role of PD-L1 stratification in optimizing health-related quality of life outcomes with pembrolizumab-based therapies in solid tumors. Across key trials, such as KEYNOTE-024, KEYNOTE-042, KEYNOTE-189, KEYNOTE-062, and KEYNOTE-048, pembrolizumab monotherapy demonstrated sustained HRQoL preservation, particularly in patients with high PD-L1 expression (e.g., ≥50% TPS), likely due to a lower burden of treatment-related toxicities compared with cytotoxic chemotherapy. In the KEYNOTE-024 trial, first-line pembrolizumab monotherapy was associated with delayed deterioration in global quality of life compared with chemotherapy in patients with PD-L1 ≥ 50% (47). In contrast, combination therapy enhances disease control in broader PD-L1 populations. Additionally, the KEYNOTE-042 study demonstrated overall survival benefits with pembrolizumab across PD-L1 TPS ≥ 1%, supporting stratified treatment approaches (48). These findings collectively reinforce the importance of PD-L1–guided treatment selection in balancing oncological efficacy with patient well-being. These patterns underscore the need for personalized treatment selection, where PD-L1 levels can guide clinicians in balancing oncologic efficacy with patient well-being. Real-world and observational studies have reported similar trends, supporting the external validity of clinical trial findings in routine practice settings.

PD-L1 expression appears to be an important biomarker influencing HRQoL trajectories. In high-PD-L1 subgroups, monotherapy was associated with better QoL maintenance, supporting guidelines recommending it as first-line therapy for NSCLC with PD-L1 expression ≥50% (49, 50). Lower PD-L1 expression levels have been associated with differential benefit from immune checkpoint inhibitor–based combination therapy, as demonstrated in phase III trials such as KEYNOTE-189 in metastatic non-squamous non–small cell lung cancer and KEYNOTE-048 in recurrent/metastatic head and neck squamous cell carcinoma, where clinical benefit with pembrolizumab-based regimens was observed across PD-L1–defined subgroups, including patients with low expression in NSCLC (TPS-based) and PD-L1–defined subgroups in head and neck cancer (CPS-based) (2, 51). This evidence supports the role of PD-L1 as a predictive biomarker for treatment selection and suggests that biomarker-informed stratification may help optimize therapeutic outcomes by aligning treatment intensity with expected clinical benefit. However, variability in PD-L1 testing methodologies, particularly differences between TPS and CPS, limits direct cross-trial comparisons and highlights the need for standardized assay implementation in clinical practice (52). In addition, different immunohistochemistry antibody assays such as 22C3 (commonly used in pembrolizumab trials), 28-8, and SP263 platforms may yield variations in PD-L1 staining intensity and positivity classification, further contributing to inter-study heterogeneity (53, 54). When combined with differences in positivity thresholds (e.g., TPS ≥ 50% vs. ≥ 1% or CPS ≥ 10), these methodological inconsistencies may lead to differences in PD-L1 subgroup classification, thereby affecting the comparability of HRQoL outcomes across KEYNOTE trials and tumor types (53, 54).

Another important consideration is that the apparent HRQoL advantage of pembrolizumab monotherapy over combination chemotherapy may also reflect differences in study populations and trial designs rather than the treatment regimen alone. Variations in baseline patient characteristics, disease burden, treatment duration, crossover, subsequent anticancer therapies, and follow-up across the included KEYNOTE trials may have contributed to differences in reported HRQoL outcomes (2, 47, 48, 50). In addition, differences in PD-L1 scoring systems (TPS versus CPS) and companion diagnostic assays (20), as well as variations in HRQoL instruments, assessment schedules, endpoint definitions, and methodological considerations for interpreting patient-reported outcomes (55, 56), further limit direct cross-trial comparisons. Therefore, the observed HRQoL differences should be interpreted cautiously, as they may reflect both treatment effects and methodological heterogeneity across studies (55–57).

An important consideration is that the observed association between PD-L1 expression and HRQoL should not be interpreted as evidence of a universal biomarker-specific effect across all solid tumors. Rather, PD-L1 primarily serves as a predictive biomarker for treatment selection, and the resulting HRQoL outcomes are likely influenced by tumor type, treatment regimen, baseline disease characteristics, and differences in PD-L1 assessment methodologies (54–56). Furthermore, the use of different scoring systems, including TPS and CPS, distinct positivity thresholds, and various companion diagnostic assays limits direct comparisons across tumor types and underscores the need for disease-specific interpretation of HRQoL findings (52, 58).

An important consideration when interpreting the current evidence is the substantial heterogeneity of HRQoL assessment across studies. Different trials employed different patient-reported outcome instruments, assessment schedules, and endpoint definitions, including HRQoL maintenance, improvement, delayed deterioration, and time to deterioration. Because these endpoints evaluate different aspects of patient experience, they should not be interpreted as equivalent measures of HRQoL benefit. These methodological considerations are consistent with current recommendations for the analysis and interpretation of patient-reported outcomes in oncology (55, 59). In addition, evidence from the included clinical trials indicates that HRQoL assessments performed during the early treatment period primarily capture acute treatment-related toxicity, whereas later assessments better reflect recovery, disease control, and long-term functional preservation.

Comparing monotherapy and combination regimens revealed trade-offs: monotherapy excelled in tolerability for PD-L1-high cases, while combinations offered survival benefits in diverse populations at the cost of initial QoL impairments. These differences may reflect the distinct toxicity profiles of immunotherapy and chemotherapy, with immune checkpoint inhibitors generally associated with fewer systemic adverse effects. Data from EORTC QLQ-C30 consistently showed symptom burden reductions with monotherapy, yet long-term responders to combinations reported QoL recovery, suggesting that patient selection and supportive care (e.g., managing toxicities) are essential. This highlights the importance of patient selection and proactive supportive care strategies to mitigate treatment-related toxicities. These insights align with broader literature, including meta-analyses of HRQoL outcomes in patients treated with immune checkpoint inhibitors (ICIs), which support the quality-of-life advantages of immunotherapy (60, 61).

### 4.2 Clinical implications

The findings from this review have several important implications for clinical practice in the management of solid tumors treated with immunotherapy. PD-L1 expression should be routinely assessed as part of standard diagnostic evaluation to guide first-line treatment selection and support a biomarker-driven, personalized treatment approach. Treatment decisions should balance expected clinical benefit with preservation of health-related quality of life. In this context, PD-L1–guided stratification can assist clinicians in selecting appropriate therapeutic strategies, where immunotherapy-based regimens may be favored in patients with higher PD-L1 expression due to their generally favorable tolerability profile and potential for better maintenance of quality of life, while combination chemo-immunotherapy may be more suitable for patients with lower PD-L1 expression requiring higher response intensity.

Although PD-L1 remains the most widely used predictive biomarker for pembrolizumab-based therapy, emerging biomarkers such as tumor mutational burden (TMB), circulating tumor DNA (ctDNA), gene-expression signatures, microsatellite instability-high/deficient mismatch repair (MSI-H/dMMR), and composite biomarker approaches may complement PD-L1 by providing additional information on tumor biology, treatment response, and prognosis, thereby supporting more precise patient selection for immunotherapy across different solid tumors (20, 62). The integration of these biomarkers with PD-L1 assessment may further improve prediction of treatment efficacy and may facilitate more individualized evaluation of long-term HRQoL outcomes, although prospective validation remains necessary. Clinicians should carefully consider the trade-off between efficacy and treatment-related toxicity, particularly during early treatment phases when symptom burden is often most pronounced. Proactive toxicity monitoring and early supportive care interventions are essential to minimize deterioration in patient-reported outcomes and maintain functional status. The integration of patient-reported outcomes into routine oncology practice is strongly recommended to enable continuous assessment of symptoms, treatment tolerability, and overall well-being. This allows timely clinical intervention and supports a more patient-centered approach to care. Finally, shared decision-making should be actively incorporated into clinical practice, taking into account PD-L1 status, baseline symptom burden, comorbidities, and patient preferences. Future prospective studies should prioritize incorporating patient-reported outcomes and HRQoL measures as primary endpoints while evaluating integrated biomarker strategies that combine PD-L1 with emerging molecular biomarkers. Standardized HRQoL assessment schedules, harmonized endpoint definitions, disease-specific biomarker frameworks, and prospective validation of integrated biomarker models will be essential to determine how precision oncology approaches influence both clinical outcomes and patient well-being (20, 55, 62). Overall, a PD-L1–guided, patient-centered treatment strategy is essential to optimize both clinical outcomes and health-related quality of life, aligning modern oncology practice with the principles of precision medicine.

### 4.3 Limitations

This narrative review has several limitations. First, although systematic reviews and meta-analyses were included, the synthesis remains qualitative and does not include new statistical pooling, which may limit the precision of comparisons between treatment strategies. Second, variability in study design, HRQoL instruments, assessment time points, endpoint definitions (including HRQoL maintenance, improvement, delayed deterioration, and time to deterioration), tumor types, and PD-L1 thresholds introduced substantial methodological heterogeneity, limiting direct comparisons across studies and requiring cautious interpretation of the relative HRQoL benefits of different treatment strategies. Third, most included studies had relatively short follow-up periods (typically 1–2 years), limiting insights into long-term HRQoL outcomes, particularly among patients with durable responses to immunotherapy. Fourth, real-world data and certain patient populations, including elderly individuals and those with multiple comorbidities, were underrepresented, restricting the generalizability of the findings.

Finally, differences in PD-L1 assessment methods, including TPS, CPS, disease-specific positivity thresholds, and companion diagnostic assays, together with inconsistent reporting of patient-reported outcomes across studies, may affect comparability and limit direct cross-tumor interpretation of biomarker-stratified HRQoL outcomes. Despite these limitations, this review provides a comprehensive synthesis of the current evidence and highlights important considerations for clinical practice and future research. Finally, differences in PD-L1 assessment methods (tumor proportion score vs. combined positive score) and inconsistent reporting of patient-reported outcomes across studies may affect comparability. Despite these limitations, this review provides a comprehensive synthesis of current evidence and highlights important considerations for clinical practice and future research.

### 4.4 Future directions

Future research should focus on prospective, PD-L1–stratified clinical studies in which HRQoL is incorporated as a primary or co-primary endpoint when comparing pembrolizumab monotherapy with combination chemotherapy. Direct head-to-head comparisons within defined PD-L1 subgroups are needed to better clarify the balance between survival benefits and quality-of-life outcomes. Standardization of PD-L1 assessment methods and HRQoL measurement tools is essential to improve cross-study comparability. Long-term follow-up evaluating sustained HRQoL trajectories is also critical, particularly in patients achieving durable responses. In addition, prospective real-world studies and registries including diverse populations, such as elderly and comorbid patients, are needed to enhance generalizability and support patient-centered precision oncology strategies.

## 5 Conclusion

This narrative review highlights PD-L1 as an important predictive biomarker for guiding pembrolizumab-based therapy; however, its interpretation is disease-specific and depends on the PD-L1 scoring system, positivity threshold, and companion diagnostic assay used for individual tumor types. Evidence from pivotal trials, including KEYNOTE-024, KEYNOTE-042, KEYNOTE-189, KEYNOTE-062, and KEYNOTE-048, indicates that pembrolizumab monotherapy in patients with high PD-L1 expression (e.g., ≥50% TPS or relevant CPS thresholds depending on tumor type) is associated with maintenance of HRQoL or delayed deterioration, consistent with lower treatment-related toxicity and durable disease control. In contrast, pembrolizumab combined with chemotherapy provides broader clinical benefit across PD-L1 subgroups but is frequently associated with an early decline in HRQoL during chemotherapy exposure due to treatment-related toxicity, followed by stabilization or partial recovery in responders. Available evidence does not suggest additional HRQoL deterioration beyond chemotherapy-related effects when pembrolizumab is added to standard regimens.

Overall, HRQoL outcomes differed across studies according to the assessment instrument, follow-up time points, and endpoint definitions, including maintenance, improvement, and delayed deterioration. These outcomes should not be interpreted as interchangeable, and direct comparisons between pembrolizumab monotherapy and combination chemotherapy should therefore be made with caution. Furthermore, distinguishing early treatment-related toxicity from long-term functional preservation is essential, as differences in HRQoL may partly reflect the timing of assessment rather than the intrinsic superiority of one treatment strategy. These findings support a biomarker-informed, patient-centered approach to treatment selection that integrates both survival outcomes and patient-reported quality of life. Future research should focus on standardized HRQoL instruments, harmonized endpoint definitions, consistent assessment schedules, standardized PD-L1 testing methodologies, and prospective PD-L1-stratified studies with long-term follow-up to further optimize precision oncology strategies.
